# Institutional experience with SRS VMAT planning for multiple cranial metastases

**DOI:** 10.1002/acm2.12284

**Published:** 2018-02-23

**Authors:** Åse Ballangrud, Li Cheng Kuo, Laura Happersett, Seng Boh Lim, Kathryn Beal, Yoshiya Yamada, Margie Hunt, James Mechalakos

**Affiliations:** ^1^ Department of Medical Physics Memorial Sloan Kettering Cancer Center New York NY USA; ^2^ Department of Radiation Oncology Memorial Sloan Kettering Cancer Center New York NY USA

**Keywords:** cranial metastases, SRS, VMAT

## Abstract

**Background and Purpose:**

This study summarizes the cranial stereotactic radiosurgery (SRS) volumetric modulated arc therapy (VMAT) procedure at our institution.

**Materials and Methods:**

Volumetric modulated arc therapy plans were generated for 40 patients with 188 lesions (range 2–8, median 5) in Eclipse and treated on a TrueBeam STx. Limitations of the custom beam model outside the central 2.5 mm leaves necessitated more than one isocenter pending the spatial distribution of lesions. Two to nine arcs were used per isocenter. Conformity index (CI), gradient index (GI) and target dose heterogeneity index (HI) were determined for each lesion. Dose to critical structures and treatment times are reported.

**Results:**

Lesion size ranged 0.05–17.74 cm^3^ (median 0.77 cm^3^), and total tumor volume per case ranged 1.09–26.95 cm^3^ (median 7.11 cm^3^). For each lesion, HI ranged 1.2–1.5 (median 1.3), CI ranged 1.0–2.9 (median 1.2), and GI ranged 2.5–8.4 (median 4.4). By correlating GI to PTV volume a predicted GI = 4/PTV^0.2^ was determined and implemented in a script in Eclipse and used for plan evaluation. Brain volume receiving 7 Gy (*V*_7 Gy_) ranged 10–136 cm^3^ (median 42 cm^3^). Total treatment time ranged 24–138 min (median 61 min).

**Conclusions:**

Volumetric modulated arc therapy provide plans with steep dose gradients around the targets and low dose to critical structures, and VMAT treatment is delivered in a shorter time than conventional methods using one isocenter per lesion. To further improve VMAT planning for multiple cranial metastases, better tools to shorten planning time are needed. The most significant improvement would come from better dose modeling in Eclipse, possibly by allowing for customizing the dynamic leaf gap (DLG) for a special SRS model and not limit to one DLG per energy per treatment machine and thereby remove the limitation on the Y‐jaw and allow planning with a single isocenter.

## 1 INTRODUCTION

Volumetric modulated arc therapy (VMAT) has significantly changed the options for LINAC based cranial stereotactic radiosurgery (SRS) treatment of multiple metastatic brain lesions. Traditionally, LINAC based SRS utilizes one isocenter for each lesion, resulting in long treatment delivery times for patients with multiple metastases. Recent publications have reported on VMAT planning for cranial SRS patients with multiple lesions using one isocenter and demonstrated that highly conformal dose distributions can be achieved.^1^, ^2^ Plan parameters have been compared to Gamma Knife plans which are considered the standard for cranial SRS treatment.^3^, ^4^ These studies have shown that VMAT plans can produce target coverage and dose fall‐off in the high dose area similar to Gamma Knife plans.

There are still challenges related to the dose accuracy of VMAT delivery for small targets and the accuracy of the VMAT dose calculation algorithm must be validated prior to releasing SRS VMAT.^5^ In addition, setup accuracy becomes much more critical when multiple targets at a distance from the isocenter are treated in the same plan.^6^

At our institution, we have developed a SRS VMAT planning technique for treatment of multiple cranial metastases using similar contouring and optimization technique to those published by Clark et al.^2^ at the University of Alabama. Due to limitations in our calculation algorithm to model both the 2.5 and 5 mm leaves on the TrueBeam STx we limit the plans to use only the 2.5 mm leaves, often resulting in two isocenters for cases with multiple brain metastasis. In this study, we present plan quality parameters and treatment times for 40 patients, treating a total of 188 lesions, with single fraction doses ranging from 16–21 Gy. The planning procedures, plan criteria, and quality assurance methods implemented at our institution are presented.

## 2 MATERIALS AND METHODS

### 2.A Preclinical dosimetry

Five VMAT test plans (total 26 lesions, 4–6 lesions each, PTV range 0.2–6.3 cm^3^) were generated in Eclipse V11.0.47 (Varian Medical Systems, Palo Alto, CA, USA) using the Progressive Resolution Optimizer (PRO). X‐ray energy of 6 MV and a dose rate of 600 MU/min were used for all plans. The plans were delivered on a TrueBeam STx (Varian Medical Systems) equipped with an MLC with 2.5 mm leaves in the center 8 cm and outer leaves of 5 mm width. For target lesions <1 cm in diameter, the Analytical Anisotropic Algorithm (AAA) photon calculation model commissioned with the gold beam data in Eclipse did not provide a dose calculation accuracy that met our departmental electronic portal imaging device (EPID) dose gamma (γ) score >95% with dose difference <3% and <2 mm distance‐to‐agreement.^5^ Therefore, a specific cranial SRS AAA (SRS_AAA) model was developed in Eclipse with source size adjusted to meet the dose agreement criteria and the test plans were re‐generated and evaluated. The 6MV AAA clinical model for a Varian TrueBeam STx was used as a baseline with focal spot (1.75 mm, 0.75 mm) and maximum field size 40 × 40 cm^2^. The dynamic leaf gap (DLG) was constrained to 1.24 mm to avoid affecting the dosimetry for the non‐SRS treatments on the same machine. Maximum field sizes, output factors, focal spot and secondary source sizes were systematically adjusted to obtain an optimized model by comparing the calculated PDD's, profiles, and outputs with water tank measurements. The source size in the fine‐tune model is (0, 0 mm). This cranial SRS_AAA model provided acceptable dosimetric agreement within the 2.5 mm leaf region, but areas with under‐dose >10% were still observed for targets treated with the 5 mm leaves.^5^ This may be a limitation of the AAA model which uses a single DLG to represent both the 2.5 and 5 mm leaves. Because of this inaccuracy, our clinical program restricts the field size of each arc in the SRS VMAT plans to the 2.5 mm leaf regions only. Consequently, one to three isocenters are required per plan depending on the spatial distribution of lesions.

Institutional plan criteria were developed prior to the clinical release of SRS VMAT based on comparing the five pre‐clinical SRS VMAT plans to the previously delivered plans developed in iPlan (RT Dose 4.5; BrainLab, Munich, Germany). The iPlan plans used one isocenter for each lesion and typically 10 static fields per isocenter. At our institution, the target dose inhomogeneity criteria for these iPlan cases were 125%. Target inhomogeneity was allowed to increase to 140% for the Eclipse SRS VMAT plans to reduce the dose to normal brain. For cases where the PTV overlapped with the brainstem, based on internal experience a dose‐volume limit to brainstem of *V*_18 Gy_ ≤ 10% was used to allow for full coverage of the target.

### 2.B Patient studies

#### 2.B.1 Immobilization and imaging

The patients were immobilized for simulation and treatment in the cranial Freedom System™ (CDR Systems, Alberta, Canada) utilizing a custom head mold and an open face mask. A triangulation point was marked on the mask using BBs at time of simulation and used for initial setup at treatment with shifts to the planner determined isocenter. Computed tomography (CT) images were reconstructed at 1.25 mm slice thickness on a Brilliance BigBore scanner (Philips Healthcare, Andover, MA, USA). Contrast‐enhanced SPGR (1 mm) and T1‐weighted (3 mm) magnetic resonance images were fused to the CT images using MIM (version 6.6.3; MIM Software Inc., Cleveland, OH, USA) and auto‐segmentation of normal structures was also generated in this systems.

#### 2.B.2 Treatment planning

The gross tumor volume (GTV) was contoured by the treating radiation oncologist who also reviewed and edited the critical structures (eyes, lenses, optic nerves, chiasm, brainstem, cord, and cochleas). A planning target volume (PTV) was created by a 3‐dimensional 0–2 mm expansion around the GTV to account for imaging fusion uncertainty, contouring variations, setup errors, and possible patient motion during treatment. The wall extraction tool in Eclipse was used to create similar shell structures for optimization as published by Clark et al.^2^ The dimension of the shells depends on the PTV size. Separate shell structures were created for each group of targets with the same prescription dose. The planner also created a structure to evaluate the GI for each PTV.

(1) $$\text{GI}=V_{50\%}/V_{100\%}$$

Depending on the spatial distribution of the lesions one, two, or occasionally three isocenters were used. The planner created a union PTV for each group of lesions that were to be treated with the same isocenter and then placed the isocenter at the geometric center of the selected group of PTVs. The isocenter was then adjusted to ensure that Y1 and Y2 jaws were ≤4 cm so that only the 2.5 mm leaves were used in order to remain within the constraints of the customized cranial SRS_AAA model. The same process was repeated for the second group of PTVs treated with the second isocenter. Each isocenter treated a distinct group of lesions. The planner selected arcs and collimator angles specific for each case to best fit the patient anatomy and distribution of lesions. For the most part, full arcs were used, however skip‐arcs were used to avoid entrance through the eyes and partial arcs were used for very lateral lesions. The planner chose the collimator angle with the goal of minimizing situations in which there were two targets in the same leaf track in order to minimize excess dose to the brain. The PRO VMAT optimizer in Eclipse V11.0.47 was used to optimize these SRS VMAT plans.

The prescribed dose to each lesion was based on lesion size and proximity to critical structures and other lesions. The initial dose volume constraints and priorities for planning follow the technique described by Clark et al.^2^ The PTV for each target was used in the optimization and the dose constrain for each PTV adjusted during the optimization so PTV *D*_98%_ for all lesions with the same prescription are as similar as possible. The plan is normalized such that all PTVs meet the coverage criteria in Table 1. The plan was normalized by setting 100% dose to cover at least 98% of the PTV volumes. Additional dose constraints were added for critical structures when needed. CI and target dose HI were used for plan evaluation:

(2) $$\text{CI}=V_{100\%}/V_{\mathrm{PTV}}$$

(3) $$\text{HI}={\text{PTV}}_{\mathrm{Dmax}}/\text{Rx}$$

**Table 1:** Institutional plan criteria, where *D*_max_ = maximum dose, *D*_min_ = minimum dose, Rx = prescription dose, and *V*_xGy_ = volume receiving x Gy

	Guideline	Limit
*Target criteria*		
PTV *D*_max_	≥125% and ≤140%	
PTV *D*_min_	≥90%	
PTV *V*_Rx_	≥98%	
*Normal tissue criteria*		
Brainstem *D*_max_	≤15 Gy	*V*_18 Gy_ ≤10% (when PTV overlap exists)
Optics *D*_max_	≤8 Gy	12 Gy
Lens *D*_max_	≤1 Gy	2 Gy
Brain *V*_7 Gy_	≤5%	
Previously treated lesions *D*_max_	≤8 Gy	
Gradient index for each lesion, PTV volume in cm^3^	≤4/PTV^0.2^	

#### 2.B.3 Patient specific quality assurance

Patient‐specific dosimetry was performed prior to treatment by scheduling and delivering the plan on an EPID. The EPID was validated to film measurements using the five preclinical plans where EPID and film measured gamma, γ, (threshold 3%/2 mm) were in agreement within 3%. Measured dose was compared to the predicted dose using the EPID module with the portal dose calculation based on the fluence calculation. A γ pass criteria for each field of 95% given a 3%/2 mm threshold was required. This was achieved for most fields with a region‐of‐interest threshold of 10%, but a threshold up to 25% was accepted where low dose was delivered though moving leaf‐gaps or closed MLC leaves to large areas. In addition, average dose over high‐dose regions of each field was assessed using the portal dosimetry histogram feature. The EPID on our TrueBeam STx at the time of this work could not accommodate flattening filter free (FFF) beams so we chose to use the standard flattened 6 MV beam for all cranial SRS VMAT plans to accommodate patient specific EPID dosimetry.

#### 2.B.4 Treatment delivery

Treatments were delivered on a TrueBeam STx with a Perfect Pitch (Varian Medical Systems) robotic 6 degree‐of‐freedom (6DOF) couch. The CDR table extension attached to the Perfect Pitch couch was used for initial adjustment of pitch and roll with guidance from the optical surface system AlignRT (VisionRT, London, UK). The AlignRT region of interest was set to cover the superior aspect of the face and forehead which was obtained from the planning CT. This process assured that any remaining pitch rotation would be within the ±3° range of the 6DOF couch. Following the initial positioning cone beam computed tomography (CBCT) images were acquired and all remaining 6D shifts were applied. Once the patient was positioned based on CBCT, a new reference AlignRT image was acquired and used with a frame rate of 2–3 Hz to detect patient motion during treatment. The beam‐off threshold set for AlignRT motion monitoring was ±1.5 mm for all translations and ±1.0˚ for all rotations.^7^, ^8^

## 3 RESULTS

### 3.A Comparison of preclinical plans

Our VMAT planning procedures were developed to produce plans comparable to our clinical standard by analyzing five clinical cases (4–6 lesions for each case) treated with iPlan plans. Comparison of plan quality parameters and brain dose for these five test cases are listed in Table 2. The brain mean dose is comparable for the two planning techniques. In our study, brain is the entire brain not excluding the targets. The CI is lower for VMAT and the GI is slightly higher. The lower GI for iPlan plans is connected to the higher CI (larger volume getting Rx dose). The volume receiving 50% of the Rx dose was smaller in the VMAT plans than in the iPlan plans for 18 of the 26 lesions, equal for six lesions, and smaller for two lesions in the iPlan plans than in the VMAT plans. A larger volume of brain received low dose (200 cGy) in the VMAT plans but a smaller volume received moderate dose (700 cGy) as compared to the iPlan plans. The resulting VMAT plan criteria for target and normal tissues are summarized in Table 1. The VMAT SRS technique is also used for patients with previously SRS treated lesions and for patients previously treated to whole brain. The maximum dose ≤8 Gy to lesions previously treated with SRS is a guide and a reminder to keep the dose as low as possible.

**Table 2:** Comparison of preclinical VMAT SRS plan with treated iPlan plans for five cases (4–6 lesions per patient)

Preclinical plans		
	iPlan	VMAT
GI	3.7 ± 0.6	4.2 ± 0.9
CI	1.4 ± 0.2	1.2 ± 0.1
Brain *D*_mean_ (Gy)	2.3 ± 0.5	2.6 ± 0.2
Brain V_2 Gy_ (%)	37 ± 6	47 ± 2
Brain *V*_7 Gy_ (%)	6 ± 3	4 ± 2

### 3.B Analysis of clinical VMAT plans

For the 40 clinical VMAT plans the number of lesions ranged from 2–8 per patient with a median of 5. The PTV volume of each lesion ranged from 0.05–17.74 cm^3^ (equivalent sphere diameter 4.6–32.4 mm) with a median value of 0.77 cm^3^, and the total tumor volume for each patient 1.09–26.95 cm^3^ (median 6.18 cm^3^). Mean and maximum dose to critical organs from the 40 treatment plans are listed in Table 3. The dose to critical structures naturally depends on the proximity to the target lesions. Higher doses were accepted for cases where the lesions were close to the critical structures and these situations are apparent in Table 3. For example, one patient had a PTV overlapping with brainstem resulting in a brainstem *D*_max_ of 24.9 Gy. For this scenario, PTV coverage was prioritized as long as brainstem *V*_18 Gy_ ≤ 10%. In another patient, an optic nerve *D*_max_ of 11.0 Gy was accepted due to the PTV proximity to the nerve. If any dose criteria were exceeded, a peer review process was initiated.

**Table 3:** Summary of doses to critical organs from 40 delivered SRS VMAT treatment plans. The number of lesions per plan ranged from 2 to 8

Critical organ	Min	Max	Median
*D*_mean_ (Gy)			
Brain	0.9	4.1	1.9
Cochlea	0.1	11.2	1.1
*D*_max_ (Gy)			
Brainstem	0.4	24.9	3.5
Chiasm	0.2	7.9	1.6
Cord	0.1	3.1	1.4
Eye	0.1	6.6	0.7
Optic nerve	0.1	11.0	1.3
Lens	0.1	1.2	0.4

**Figure 1:**
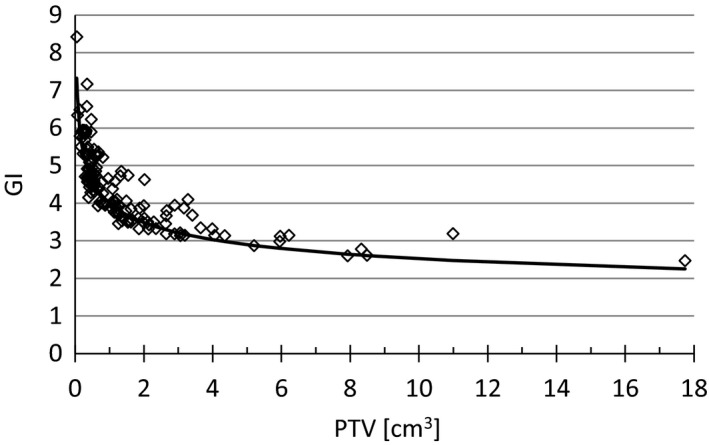
Gradient index (GI) as a function of target volume of each lesion. The solid line is the function 4/PTV^0.2^ which, as a result of this analysis, was implemented to calculate the expected GI depending on the PTV volume in cm^3^ for each individual lesion during planning.

**Figure 2:**
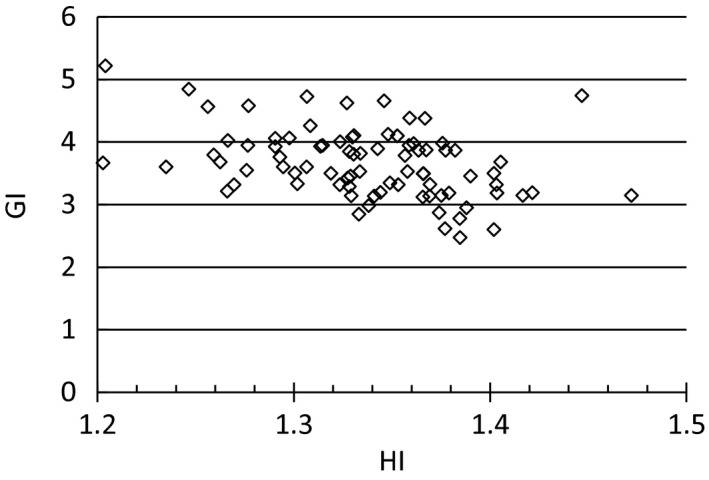
Gradient index (GI) as a function of the dose heterogeneity index for all PTVs ≥0.8 cm^3^. For these lesions there is slight reduction in GI with increasing HI. For lesions smaller than 0.8 cm^3^ this trend was not observed.

The target indexes GI, HI, and CI were collected for each PTV. The CI is typically in the range 1.0–1.2. The maximum value of 2.9 occurred for a 0.05 cm^3^ lesion in a 6‐lesion plan. The median GI is 4.4. Figure 1 shows the GI for each lesion plotted versus PTV volume. Out of the 188 lesions, 13 lesions were so close that the 50% isodose was not split between the two lesions. These lesions were excluded from this GI analysis. For PTV sizes >0.5 cm^3^, a GI <5 is typically achievable whereas for smaller targets, GI exceeds 5. The GI is reduced with increasing PTV size. Allowing the HI to increase facilitates a slight reduction in the GI for lesions ≥0.8 cm^3^ as seen in Fig. 2. This trend was not observed for the smaller lesions.

**Figure 3:**
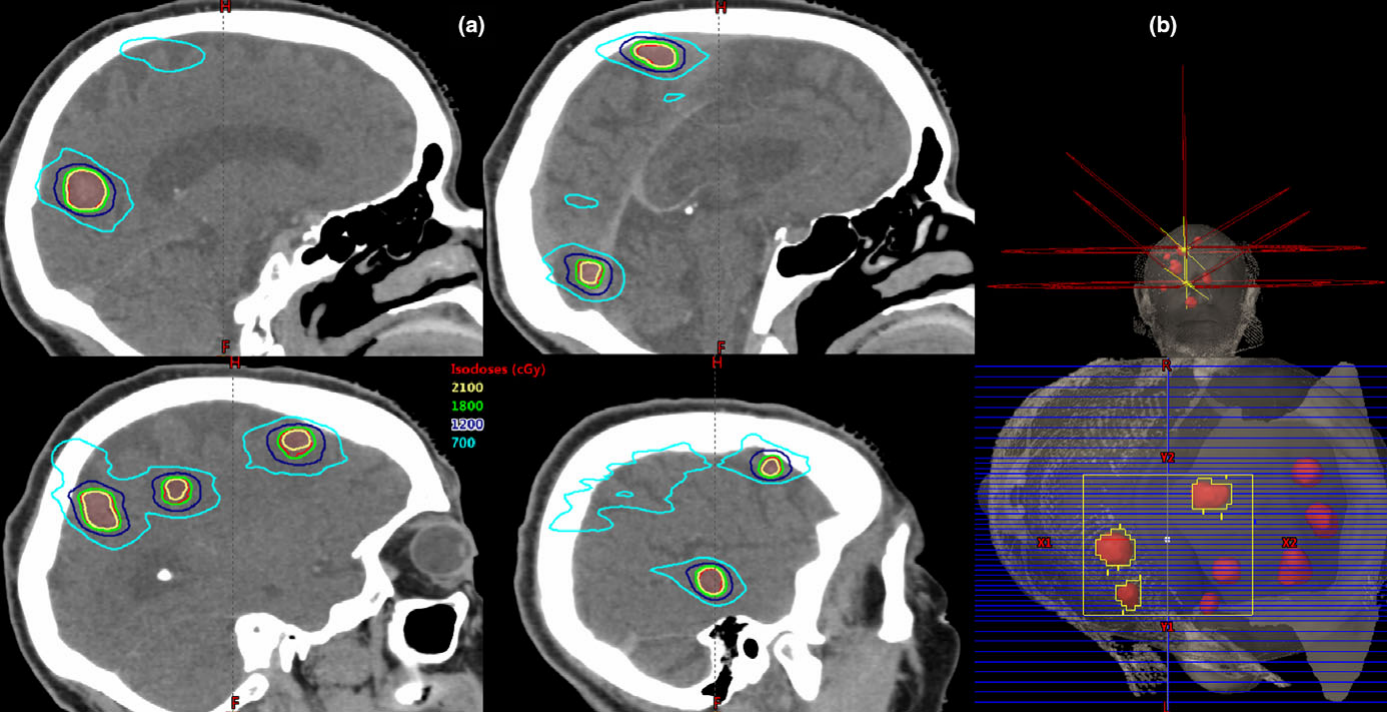
(a) Isodose distributions for an eight‐lesion case. Three lesions received 21 Gy and five lesions received 18 Gy. The total tumor volume was 15.4 cm^3^. (b) Two isocenters were used with five arcs on each isocenter. The couch angles for both isocenters were the same: 0, 0, 40, 90, and 330. The collimator example is for one arc in the superior isocenter treating three lesions. The collimator for each arc was manually selected with the goal of minimizing situations in which there were two targets in the same leaf track in order to minimize excess dose to the brain.

Isodose distributions for a typical plan with eight lesions and two isocenters are shown in Fig. 3(a). For this case the total PTV volume was 15.4 cm^3^ with lesions ranging from 0.67–3.16 cm^3^. The CI was 1.1 for four of the lesions, 1.2 for one lesion and 1.3 for three lesions. The GI ranged from 3.2 to 5.3, with the highest value for the smallest lesion. The following doses to critical structures were achieved: brain *D*_mean_ = 3.8 Gy, brain *V*_7 Gy_ = 8.9%, brainstem *D*_max_ = 4.3 Gy, lenses *D*_max_ < 0.7 Gy, and *D*_max_ to all other optical structures <2.5 Gy. Figure 3(b) shows the arcs used for this case. Two isocenters were used with five arcs at couch 0, 0, 40, 90, and 330 for each isocenter. The collimator for each arc was manually selected to minimizing situations in which there were two targets in the same leaf track.

**Figure 4:**
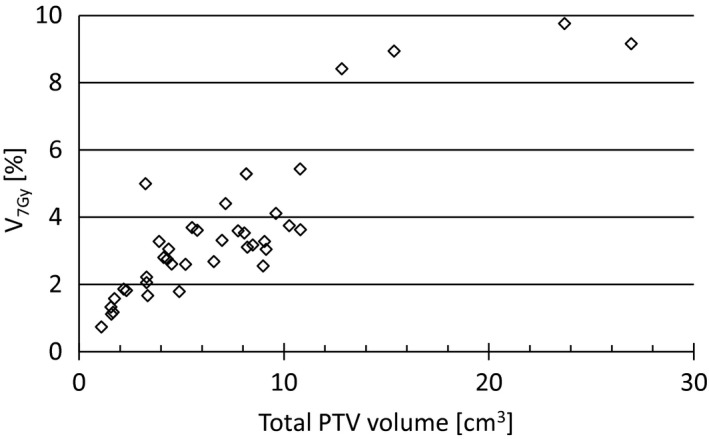
Percent brain volume receiving more than 7 Gy as a function of the total PTV volume (sum of all PTVs for each patient). The plan goal is to achieve brain *V*_7 Gy_ < 5%.

The percent brain volume receiving 7 Gy or less is plotted in Fig. 4 as a function of the total tumor volume (sum of all PTVs) in each treatment plan. The plan goal is to keep brain *V*_7 Gy_ < 5% which is a criteria based on internal experience. For cases with a large tumor burden this criteria was not achieved.

Total beam‐on time for each patient and beam‐on time per isocenter is listed in Table 4 along with the total treatment time for each patient measured from the first CBCT to completed delivery and the total treatment time per isocenter. Ten plans used one isocenters, 28 plans used two isocenters, and two plans used three isocenters. The setup time prior to the first CBCT is not included since this is not recorded in the record and verify system. By analyzing the AlignRT data we found that typically it takes 5 min from when AlignRT is turned on for manual adjustment of the CDR board until the 1st CBCT is acquired. For all cases only one CBCT was needed since the manual adjustment of the CDR board with AlignRT guidance assured that the patient position was within the range of the 6D couch.

**Table 4:** Beam‐on time for each patient and per isocenter, and total treatment time for each patient and per isocenter, for the 40 plans. Median treatment time per isocenter is 31 min. Ten plans used 1 isocenters, 28 plans used 2 isocenters, and 2 plans used 3 isocenters

	Time (min)		
	Min	Max	Median
Beam‐on total	9	33	19
Beam‐on per isocenter	9	17	11
Treatment total	24	138	61
Treatment per isocenter	24	48	31

To determine the percentage of patients that moved and were repositioned during treatment off‐line review sessions for 240 single fraction cranial patients were reviewed. We found that 10 patients (4.2%) were repositioned during treatment. One patient was repositioned three times. Eight patients moved <1.1 mm (root‐mean‐square of shifts in all three translation directions), one patient moved 1.8 mm, and one patient moved 6.8 mm. The largest rotational changes were in the pitch angle with up to 1.5°, up to 1.4° in roll, and up to 0.6° in rotation.

## 4 DISCUSSION

Several studies have compared the dose distribution and dosimetric parameters for Gamma Knife plans to VMAT plans for cases with multiple metastases. One study reported that VMAT plans provide superior conformity with no significant difference in dose fall off compared to Gamma Knife plans,^4^ whereas another study found that VMAT plans had comparable conformity to Gamma Knife but that Gamma Knife remained superior in terms of dose fall off around the target.^9^ Liu et al. found that VMAT plans provide better conformity but a larger GI than Gamma Knife, that moderate to low dose (3–6 Gy) isodose volumes were equivalent to Gamma Knife, and that Gamma Knife achieved smaller low‐dose (<3 Gy) volumes.^3^ The differences in GI will depend on the VMAT planning technique as well as on the size of the lesions, so a direct comparison between studies at different institutions is not necessarily possible. Our results comparing the VMAT plans to the delivered 3D plans using 10–12 static fields using a single isocenter in each lesion concur with the results from Liu et al. that VMAT plans result in larger brain volumes receiving low dose, but moderate to intermediate dose is lower in the VMAT plans, and VMAT can achieve better CI. Thomas et al. reported results for cases with 2–9 lesions and total tumor volumes from 0.23–19.56 cm^3^, all planned with 18 Gy prescriptions.^4^ Our preclinical cases had 2–8 lesions with a total tumor volume ranging from 1.09 to 26.95 cm^3^ and most of the lesions were prescribed 21 Gy. The larger total tumor volume in our study and the higher prescription dose resulted in a larger mean brain dose (range 93–413 cGy) as compared to brain mean dose up to 200 cGy in their study. Clark et al. published a mean GI of 3.34 ± 0.42 for 15 VMAT plans with 1–5 targets with size range from 0.67–44.68 cm^3^.^2^ In our study the GI range 2.5–8.4 with a mean of 4.4. Our results show that the GI is reduced with increasing target size so it is therefore expected that our reported GI is higher than in Clarks study where the lesion sizes were larger.

Ma et al. published a planning study comparing plans for Gamma Knife, Cyber Knife, Novalis, and TrueBeam FFF for cases with 3, 6, 9, and 12 lesions, concluding that the volumes of brain receiving low to moderate dose (4–12 Gy) were higher and increased more rapidly with additional targets for LINAC‐based SRS than for Gamma Knife.^10^ In this study, all lesions were smaller than 1 cm^3^, whereas in our study the targets ranged from 0.05–17.74 cm^3^. Therefore, the brain volume receiving 12 Gy is higher in our study (range 4–57 cm^3^, median 19 cm^3^) than the TrueBeam results in their study (increasing from 5.5 to 29.6 cm^3^ as the lesion number increase from 3 to 12).

From the preclinical VMAT planning we found that when increasing the HI while keeping the CI the same, the GI was reduced (data not shown). Other groups have reported reduction in GI with increasing HI.^11^, ^12^ GI as a function of the dose HI for all PTVs ≥0.8 cm^3^ is shown in Fig. 2. There is no strong correlation between GI and HI from these clinical plans but there may have been different considerations or challenges in the plans that impacted the final HI and GI for each lesion. We decided to allow for a PTV *D*_max_ of 140%. This is higher than for the iPlan plans that have been our clinical standard prior to introducing the VMAT technique but since Gamma Knife plans typically use a PTV *D*_max_ >140% we accepted this higher inhomogeneity to reduce the GI. The risk of radiation necrosis following cranial SRS has been reported in several studies.^13^, ^14^, ^15^, ^16^, ^17^, ^18^ More data are needed to determine if the difference in dose distribution between the planning techniques have potential clinical consequences.

Following the analysis of these treatment plans, we have implemented a GI criterion that more closely follows the expected fall‐off with increasing lesion size, Fig. 1. The solid line in this plot is the empirical function 4/PTV^0.2^ where PTV is the planning target volume for each individual lesion. This function has been built into a plan evaluation tool by creating a script in Eclipse that our planners can use during planning to calculate expected GI for each lesion.

Prior to developing the cranial SRS VMAT procedure, a 3D technique using static MLC fields in iPlan with one isocenter in each lesion was used for SRS planning at our institution. For cases with three or more lesions both planning time and treatment time were significant. The motivation to treat multiple cranial lesions with VMAT using one or two isocenters is based on a significant reduction in treatment time. The median beam‐on time per isocenter is 11 min and the median treatment time per isocenter is 31 min for the 40 clinical VMAT cases presented in this study. The difference in these two times is the time it takes to acquire the CBCT, get physician verification, and rotate the couch to planned angles. Typical time from setup to end of treatment for a single lesion iPlan SRS case is 20–30 min which is similar to the treatment time per isocenter for the VMAT cases. For cases with three or more lesions, the treatment time is significantly reduced by using VMAT with one or two isocenters. Planning time for one or two lesion cases are significantly shorter for a 3D plan in iPlan than for VMAT in Eclipse. Inverse planning requires contouring of optimization structures and running at least one optimization. Contouring time can be reduced by utilizing automatic segmentation of critical structures. We are using atlas auto‐segmentation in MIM and a specifically designed workflow to create optimization structures for each VMAT case. Isocenter position and arcs are placed once the CT and structure set is transferred to Eclipse. Due to limitations in the dose modeling we limit our plans to the 2.5 mm leaves on the TrueBeam STx. This limitation significantly increases the planning time since placement of the isocenters to best target all lesions is not trivial. The planner will group lesions based on the spacial distribution with the criteria that all lesions in the group can be treated with one isocenter and using only the small MLC leaves. Currently this is a manual process. The planner will place the isocenter at the geometric center of the union PTV and then evaluate if all lesions can be targeted with their chosen arcs. The isocenter may be adjusted to ensure Y1 and Y2 jaws ≤4 cm to remain within the constraints of the customized cranial SRS_AAA model.^5^ Each isocenter is treating a single, distinct group of lesions. For cases where the lesions naturally are grouped this process is easier than when all lesions are spread evenly thought the brain. Placing isocenters and selecting arcs takes 1–2 h for a 10 lesion case. Running the optimization in Eclipse for two separate plans takes up to 2 h. Adding time for fusion and contouring, the total planning time for a ten lesion SRS VMAT case can be up to 5 h.

The reduction in treatment time with VMAT facilitates treatment of multilesion SRS cases on a LINAC but new developments in planning software are needed to reduce the significant planning time. The lack of accurate modeling for both MLC types on the TrueBeam STx does introduce limitation that complicates planning. We investigated a new AAA model for a TrueBeam M120 (Varian Millennium 120 MLC with 5 mm MLC in the central 20 cm × 20 cm field) to determine if we could overcome the limitation by using a machine with only one type of MLC leaves in a field large enough to cover the entire brain but for the 5 mm leaves we found that the dose inaccuracy was unacceptable for lesions with diameter <10 mm (data not shown). For cases with multiple metastatic lesions this scenario is unrealistic. Better dose modeling in Eclipse that provides accurate calculation for both MLC types would remove the limit on the Y‐jaws and allow planning with a single isocenter. This would significantly reduce planning and treatment time. In the current situation with lack of accurate dose modeling, software tools can be developed to assist with optimal grouping of targets into separate isocenters and plans that use only the 2.5 mm MLC leaves.^19^ Similarly to what we found for the AAA dose calculation model Gardner et al. reports the need to adjust source size for small field intracranial SRS using AcurosXB in the Eclipse planning system to avoid >10% central axis dose discrepancies for small target volumes.^20^

When treating multiple targets distant from the isocenter, extra requirements are needed for setup accuracy and limitation of patient motion during treatment. To remove any patient rotation at setup, a 6DOF couch should be used for treatment. The positioning accuracy is determined by the CBCT‐MV isocenter congruence, the amount of couch walk and the accuracy of the image registration at the machine. Motion monitoring with conventional LINAC on‐board imaging is challenging due to limitations imposed by the routine use of couch rotations and gantry rotation for these patients. Using the optical surface image system AlignRT we found that few patients move during treatment in our frameless immobilization system. Most of the patients that moved (eight out of ten) had moved <1.1 mm but there were a couple of patients that moved significantly and this highlights the importance of using a motion monitoring system to catch these outliers. An alternative motion monitoring system frequently used for cranial SRS is ExacTrac (Brainlab, Munich, Germany).

## 5 CONCLUSION

We have developed a procedure to treat multiple cranial metastases with VMAT achieving similar plan quality to traditional 3D LINAC based SRS plans. Caution must be taken to assure that the dose calculation model is accurate for very small lesions and for both MLC types on a TrueBeam STx. For cases with three or more lesions treatment time is significantly reduced by using VMAT plans and one or two isocenters. Currently it is very elaborate to create these treatment plans due to limitations in the dose modeling, and also due to contouring and optimization. Better dose modeling in Eclipse would remove limitations on the Y‐jaw and thereby reduce planning and treatment time significantly.

## CONFLICT OF INTEREST

There is no conflict of interest related to this study.
